# *Rehmannia glutinosa* Extracts Enhances Ketone Bodies Expression and Alleviates Ulcerative Colitis as A Novel Ketogenic Functional Food

**DOI:** 10.1002/fsn3.72263

**Published:** 2026-08-16

**Authors:** Yiqiao Gao, Simiao Wang, Yuye Yang, Wei Tang, Jianhan Zhen, Xiangshi Song

**Affiliations:** ^1^ School of Pharmacy Henan Medical University Xinxiang China

**Keywords:** anti‐inflammatory effect, ketogenic diet, ketogenic functional food, *Rehmannia glutinosa*, STAT3 pathway, ulcerative colitis

## Abstract

The ketogenic diet (KD), as a clinically common method to increase ketone bodies, can induce adverse reactions due to alterations in normal dietary structure. This study aims to elucidate that *Rehmannia glutinosa* (DH), a representative plant among the “medicinal and edible homologous traditional Chinese medicines”, also exhibits ketogenic effects and can be utilized to alleviate ulcerative colitis (UC). The ketogenic effects of DH were confirmed through molecular docking, enzyme activity assays, energy metabolism indicators, and ketone levels measurements. The safety profiles of DH were evaluated through hepatic and renal function indicator tests. The effects of DH and the traditional KD on UC were validated and compared via a dextran sulfate sodium‐induced C57BL/6J mouse model and the measurement of pathological indicators related to UC. Animal studies demonstrated that administering DH extracts at a dose of 1.5 g/kg/day effectively enhanced ketone bodies expression in mice. This effect might be attributed to the increased activity of key enzymes involved in ketogenesis, such as 3‐hydroxybutyrate dehydrogenase 1 and 3‐hydroxymethylglutaryl‐CoA synthase 2. In the UC model, both DH and KD significantly alleviated intestinal inflammation, oxidative stress injury, and mitigated UC damage. The primary mechanism might be related to the suppression of the STAT3 pathway. This study demonstrates that DH can serve as a novel ketogenic functional food to enhance ketone bodies expression and be applied for UC management.

## Graphical abstract

*Rehmannia glutinosa* (DH) can act as a novel ketogenic functional food to enhance the expression of ketone bodies without altering the normal dietary structure. This effect might be attributed to the increased activity of key enzymes involved in ketogenesis, such as 3‐hydroxybutyrate dehydrogenase 1 (BDH1) and 3‐hydroxymethylglutaryl‐CoA synthase 2 (HMGCS2). The dual‐action synergy of DH combines direct phytochemical anti‐inflammation with secondary ketogenic effects, presenting potential therapeutic applications for UC. The potential mechanism of DH in mitigating UC may be related to the inhibition of STAT3 phosphorylation.

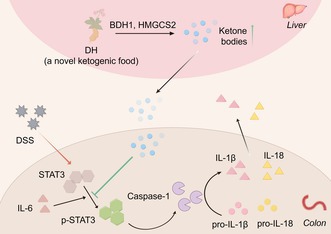

## 1 Introduction

The ketogenic diet (KD) is a dietary regimen distinguished by its low carbohydrate intake and high fat content, which forces the liver to metabolize fats into ketone bodies as the primary energy source (Meng et al. 2024). The ketogenic diet has been applied in the prevention and treatment of conditions such as epilepsy and cancer (Neal et al. 2008; Qin, Huang, et al. 2024). However, due to the alteration of normal dietary patterns, the ketogenic diet may cause adverse effects such as fatigue, diarrhea, and ketoacidosis, and long‐term use may lead to hepatic, renal, and cardiovascular diseases (Javier et al. 2024; Meng et al. 2024). Therefore, a novel ketogenic approach without modifying the normal dietary structure holds greater potential for development.

The major mechanism of the KD is that it can increase the level of ketone bodies. Ketone bodies are a class of energy sources distinct from glucose, primarily synthesized within the mitochondria of hepatic cells. Ketone bodies include acetoacetate, β‐hydroxybutyrate (βOHB), and acetone, with βOHB comprising approximately 70% of the total ketone bodies (Puchalska and Crawford 2021). Numerous research has elucidated that βOHB can act as an anti‐inflammatory signaling molecule. It mitigates inflammatory responses by limiting potassium ion efflux, inhibiting the expression of inflammatory mediators, and other related pathways (Yao et al. 2021; Youm et al. 2015). Presently, ketogenic therapies leverage the anti‐inflammatory benefits of ketone bodies for both preventing and treating inflammation‐related issues, including ulcerative colitis (UC) (Luiskari et al. 2024; Suzuki et al. 2023).

UC is a long‐term inflammatory bowel disease characterized by diffuse inflammation affecting the colon and rectum. The worldwide occurrence of UC has been steadily increasing in recent years, establishing it as one of the primary clinical digestive disorders (Berre et al. 2023). The primary clinical manifestations of UC include diarrhea, rectal bleeding, and severe abdominal pain. Although the precise etiology of UC remains unclear, it is widely accepted that the condition arises from a multifactorial interplay involving immune dysregulation, alterations in gut microbiota, genetic predisposition, and environmental influences (Gros and Kaplan 2023). Currently, the common pharmacological treatments for UC include aminosalicylic acids, corticosteroids, and immunomodulators (Liang et al. 2024; Wangchuk et al. 2024). Nonetheless, these treatments frequently come with considerable difficulties, including adverse reactions, poor overall efficacy, hormone dependence, and drug resistance (Hashash et al. 2025; Liang et al. 2024). Given that ketogenic therapies have been applied in UC treatment, developing a safer ketogenic approach could be a novel strategy for UC management.

The theory of “Chinese food‐medicine homology”, which posits that food and medicine share commonalities in their origins and therapeutic effects, has gained international recognition (Liu, Zhou, et al. 2025). Recent studies have shown that medicinal and edible homologous traditional Chinese medicines (MEH) are uniquely advantageous in preventing and treating diseases through the multi‐active components and multi‐target mechanisms, offering benefits such as immunity regulation, improvement of gut microbial balance, and repair of intestinal mucosal impairment (Liu et al. 2022). With fewer adverse reactions and better patient tolerance, MEH shows great potential for development as a functional food to provide superior therapeutic options for UC, making it a key focus in related research (Cheng et al. 2024; Liu, Yang, et al. 2025).

*Rehmannia glutinosa* (Gaertn.) Libosch (DH) is a prominent MEH of the family Scrophulariaceae (Jia et al. 2023). The common edible forms of DH include fresh DH, dried DH, prepared DH, and so on (Bian et al. 2023). As a representative of MEH, DH is distinguished by its easy administration, favorable safety profiles, and strong patient compliance in application (Li et al. 2025; Zhang et al. 2025). Investigations have revealed that DH may exert anti‐inflammatory effects through pathways such as NF‐κB and Bcl‐2/Bax (Bian et al. 2023; Jia et al. 2023). However, apart from directly acting on disease targets, recent studies have demonstrated that MEH can also exert therapeutic effects by modulating the metabolism of endogenous functional molecules, such as βOHB (Chen et al. 2023; Gao, Yang, et al. 2025).

In numerous disorders, such as heart failure and Parkinson's disease, βOHB has been shown to inhibit signal transducer and activator of transcription 3 (STAT3) and cysteine‐requiring aspartate protease 1 (caspase‐1), thereby alleviating pyroptosis (Deng et al. 2021; Jiang et al. 2022). STAT3, a multifunctional transcription factor, is pivotal in regulating cellular processes, including growth, differentiation, proliferation, apoptosis, and immune responses (Wen et al. 2024). STAT3 can promote the activation of caspase‐1, a crucial protein in innate immunity. Caspase‐1 activation subsequently promotes the rapid expression of IL‐1β and IL‐18, thereby inducing the inflammatory response (Feng et al. 2022). Numerous studies have established that the targeted modulation of STAT3 has emerged as a significant research focus in the therapeutic intervention of UC and other related inflammatory disorders (Huang et al. 2024; Xue et al. 2026). However, whether the ketogenic effects of DH or KD can alleviate UC by inhibiting STAT3 remains unexplored.

This study hypothesized that DH, in addition to its inherent anti‐inflammatory and antioxidant properties attributed to its active components, can also serve as a novel ketogenic functional food by enhancing the expression of ketone bodies, such as βOHB. This dual‐action synergy combines direct phytochemical anti‐inflammation with secondary ketogenic effects, presenting potential therapeutic applications for UC. To validate this hypothesis, this study initially employed molecular docking, enzyme activity assays, and animal experiments to confirm that DH exhibits ketogenic effects. Subsequently, using a dextran sulfate sodium (DSS)‐induced C57BL/6J mouse UC model, we demonstrated that DH can achieve effects similar to those of the traditional KD, both of which effectively mitigate colitis damage, with the mechanism potentially involving inhibition of the STAT3 pathway. Meanwhile, compared to the traditional KD, DH demonstrates superior antioxidant, anti‐inflammatory, and safety characteristics in the UC model. These findings indicate that DH can act as a novel ketogenic functional food, enhancing the expression of ketone bodies without altering normal dietary structure and may be applied for UC management.

## 2 Materials and Methods

### 2.1 Materials and Reagents

Prepared DH decoction pieces (origin geographical location: Went County, Henan province of China, specimen number: 230708‐1) were acquired from Zhangzhongjing Pharmacy (Henan, China). The specimens were stored at Henan Medical University and identified by Dr. Gao. DSS (M.W. 36,000–50,000) was obtained from MP Biomedicals (USA). Mesalazine (5ASA, purity: > 99%) was acquired from Aladdin Biochemical Technology Co. Ltd. (Shanghai, China). Herbal reference standards, including catalpol (purity: 99.53%) and ajugol (purity: 99.82%) were obtained from Herbpurify Co. Ltd. (Chengdu, China). The KD and standard diet for mice were sourced from Sibeifu Biology Technology Co. Ltd. (Beijing, China), and the macronutrient composition is detailed in Table S1.

A designated procedure was used to prepare the DH extracts. The prepared DH decoction pieces (50 g) were soaked in distilled water (300 mL) for 2 h before being boiled at 100°C for 1 h. Following filtration, the residue was re‐decocted with distilled water (200 mL) for 1 h. The resulting decoctions were combined and concentrated to a total volume of 100 mL and stored at 4°C for later application.

### 2.2 Quality Control of DH Extracts

The data on characterization (utilizing chromatographic methods) and the quantitative control of the primary active compounds are provided in the Supporting Information.

### 2.3 Molecular Docking

The identification of active chemical components in DH was conducted using the TCMSP Database (https://www.tcmsp‐e.com) and the PUBCHEM database (https://pubchem.ncbi.nlm.nih.gov). Target proteins involved in ketone bodies metabolism were identified based on existing literature and KEGG (https://www.kegg.jp/). The 3D structures of these target proteins were obtained from the RCSB PDB database (https://www.rcsb.org/). PyMOL software (Version 2.2.0) was employed to remove solvent molecules, followed by the use of AutoDock Tools (Version 1.5.7) for hydrogenation, charge assignment, and calculation of the minimum binding energy between the active chemical components and the target proteins. The docking outcomes for the target proteins and active chemical components with the minimum binding energy were chosen and visualized using PyMOL software (Version 2.2.0).

### 2.4 Animal Experiments

The animal experimental procedures were approved by the Animal Ethics Committee of Henan Medical University (XYLL‐20240321), in accordance with the Guide for the Care and Use of Laboratory Animals. Forty‐eight C57BL/6J mice (male, 6–8 weeks, SPF grade, weighing 22 ± 3 g) were obtained from Henan Skobes Biotechnology Co. Ltd. (SCXK‐2024‐005). The animals were housed in a temperature and relative humidity controlled environment under a 12 h dark/light cycle at the Animal Experiment Center of the School of Pharmacy, Henan Medical University (Xinxiang, China). Following 1 week of adaptive rearing, the mice were randomly divided into six groups using a random number table generated in Microsoft Excel 2021: control (*n* = 8, C), UC model (*n* = 8, DSS), high‐dose DH extracts (*n* = 8, DSS + DHh), low‐dose DH extracts (*n* = 8, DSS + DHl), mesalazine (*n* = 8, DSS + 5ASA), and ketogenic diet (*n* = 8, DSS + KD). An a priori power analysis (G*Power 3.1.9.7) was performed to justify the sample size.

The DSS‐induced UC mouse model was developed in accordance with established methodologies from the literature. Except forthe C group, mice in other groups were administered 3% (w/v) DSS in drinking water ad libitum for seven consecutive days (Days 1–7) to induce UC. As a commonly used medication for UC, mesalazine (5ASA) was used as the positive control drug. Standardized protocols for administering DSS and 5ASA were rigorously followed throughout the experiment (Cheng et al. 2021; Li et al. 2024).

Based on existing literature and preliminary studies (Gao, Wang, et al. 2025), the dosages of DH extracts were established at 1.5 g/kg/day and 0.75 g/kg/day, respectively. From Days 1 to 7, the DSS + 5ASA (200 mg/kg/day), DSS + DHh, and DSS + DHl groups were administered their respective medications through oral gavage, and the C group was given saline using the same method. Throughout the experiment, mice in the DSS + KD group were fed a traditional KD, whereas those in other groups were provided with a standard diet. At the end of the experiment on Day 8, mice were euthanized, and samples of blood, liver, spleen, and colon tissues were collected for subsequent examination.

### 2.5 Disease Activity Index Scoring and Spleen Index

The Disease Activity Index (DAI) of each mouse was calculated to assess the severity of UC. The DAI was calculated based on parameters such as weight loss, fecal characteristics, and hematochezia (Kihara et al. 2003). Detailed evaluation criteria are provided in Table S2. The spleen index was computed by the formula: spleen index = spleen weight (g)/body weight (g).

### 2.6 Histopathological Examination

The colon tissues were fixed in 4% paraformaldehyde and subsequently embedded in paraffin following standard protocols. The sections embedded in paraffin were stained with hematoxylin and eosin (H&E) and examined using confocal microscopy (Olympus FV1200, Japan) to assess morphological and pathological changes in the colon. Detailed criteria for the quantitative blinded histopathological score are provided in Table S3 (Kihara et al. 2003).

### 2.7 Enzyme Activity Assays

Liver tissues were homogenized on ice in PBS using a tissue grinder (Jingxin JXFSTPRP‐48L, China). Tissue homogenates and plasma samples were centrifuged (4°C, 3000 *g*, 10 min), and the supernatant was collected. Using activity detection kits (Solarbio, Beijing, China), the spectrophotometric absorbance changes of enzymatic reaction products or their derivatives were measured (Molecular Devices SpectraMax i3x, USA) after incubation at a constant temperature for the specified duration. The enzymatic activities of 3‐hydroxybutyrate dehydrogenase 1 (BDH1) and 3‐hydroxymethylglutaryl‐CoA synthase 2 (HMGCS2) in the liver, as well as alanine aminotransferase (ALT) and aspartate aminotransferase (AST) in plasma, were subsequently calculated.

### 2.8 Biochemical Examination

Colon tissues were homogenized with PBS utilizing a tissue grinder (Jingxin JXFSTPRP‐48L, China). Tissue homogenates and plasma samples were centrifuged (4°C, 3000 *g*, 10 min), and the supernatant was collected for biochemical examination. Total protein levels in the supernatant were tested using the BCA protein assay kit (Solarbio, Beijing, China). The levels of total ketone bodies, βOHB, insulin, BDH1, HMGCS2, myeloperoxidase (MPO), IL‐6, p‐STAT3, STAT3, caspase‐1, TNF‐α, IL‐18, and IL‐1β were quantified using ELISA kits (Meilian Biotechnology Co. Ltd., Shanghai, China). Plasma glucose, triglycerides (TG), creatinine, blood urea nitrogen (BUN), and uric acid were measured using assay kits (Solarbio, Beijing, China). Assays for total free sulfhydryl groups (T‐SH), malondialdehyde (MDA), superoxide dismutase (SOD), and total antioxidant capacity (T‐AOC) were conducted using the specific assay kits (Beyotime Biotechnology, Shanghai, China).

### 2.9 Statistical Analysis

Statistical analyses and graphical representations were performed using SPSS (version 32.0) and GraphPad Prism (version 10.4). The pathway was visualized by Figdraw 2.0. Normality and homogeneity of variance tests were conducted on the data. If the parametric assumptions were satisfied, one‐way ANOVA was employed with Tukey's post hoc test for multiple‐group comparisons; if not, the Kruskal–Wallis H test was used for overall non‐parametric comparisons among multiple groups, followed by Dunn's post hoc test for pairwise comparisons. A *p*‐value under 0.05 indicated statistical significance. Correlation analysis was conducted using Spearman's analysis, and a correlation was considered significant when the significance value of the correlation coefficient was less than 0.05. The experimental results are presented as mean ± standard deviation (*n* = 8 per group).

## 3 Results

### 3.1 Molecular Docking Between Active Components of DH and Ketogenesis‐Related Proteins

**FIGURE 1:**
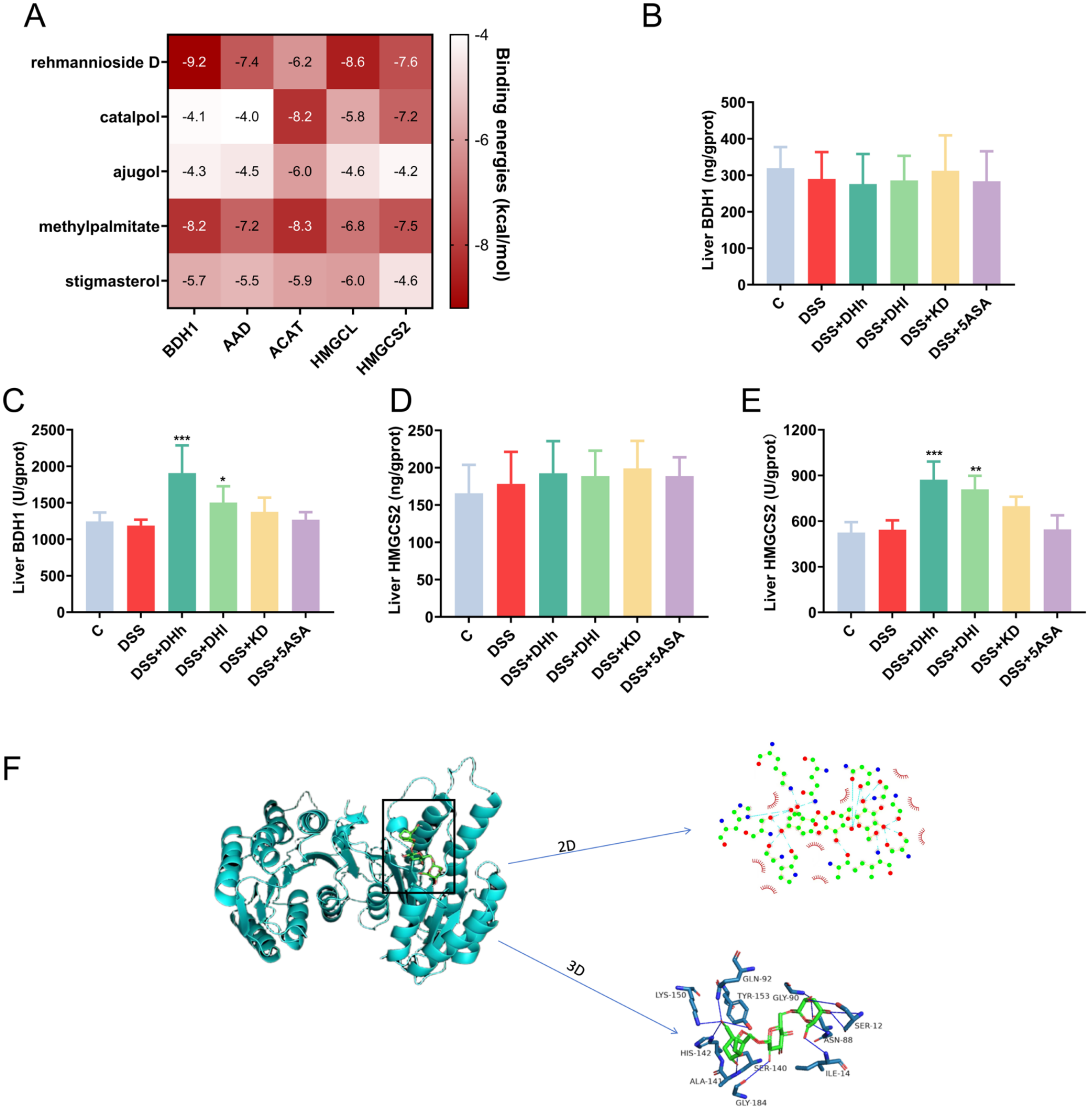
Molecular docking results and preliminary validation. (A) Heat map of the binding energies between the main active components of DH and the target proteins; Mice liver BDH1 expression levels (B, C) on Day 8; Mice liver HMGCS2 expression levels (D, E) on Day 8; (F) Representative molecular docking diagram of rehmannioside D with BDH1 (green, red, and blue: Carbon atom, oxygen atom, and nitrogen atom). ***p < 0.001, **p < 0.01, *p < 0.05 compared to the DSS group.

To explore the potential ketogenic properties of DH, we utilized molecular docking techniques for preliminary validation. Molecular docking simulations were performed between the primary active components of DH identified from the database and the proteins related to ketogenesis. Binding energies were calculated and are presented in Figure 1A. The results indicated that certain active components in DH exhibited strong binding affinities with the chosen protein targets, including BDH1, acetoacetate decarboxylase (AAD), acetyl‐CoA‐acetyltransferase (ACAT), 3‐hydroxymethylglutaryl‐CoA lyase (HMGCL), and HMGCS2. The UniProt IDs of these protein targets are presented in Table S4. To preliminarily validate the molecular docking results, we selected BDH1 (which exhibited the lowest binding energy) and HMGCS2 (the key rate‐limiting enzyme in ketogenesis) (Puchalska and Crawford 2021), and measured their expression levels and activities in the liver. The results demonstrated that DH did not significantly affect the expression levels of BDH1 or HMGCS2, but significantly enhanced their enzymatic activities (Figure 1B–E). These findings suggest that in the DSS‐induced UC model, DH may influence the production of ketone bodies by regulating the activities of enzymes involved in hepatic ketogenesis.

### 3.2 Ketogenesis of DH in Comparison to the Traditional KD

The quantitative control results for DH extracts are presented in Table S5 and Figure S1. The DH extracts were consistently utilized in the subsequent experiments without replacement.

**FIGURE 2:**
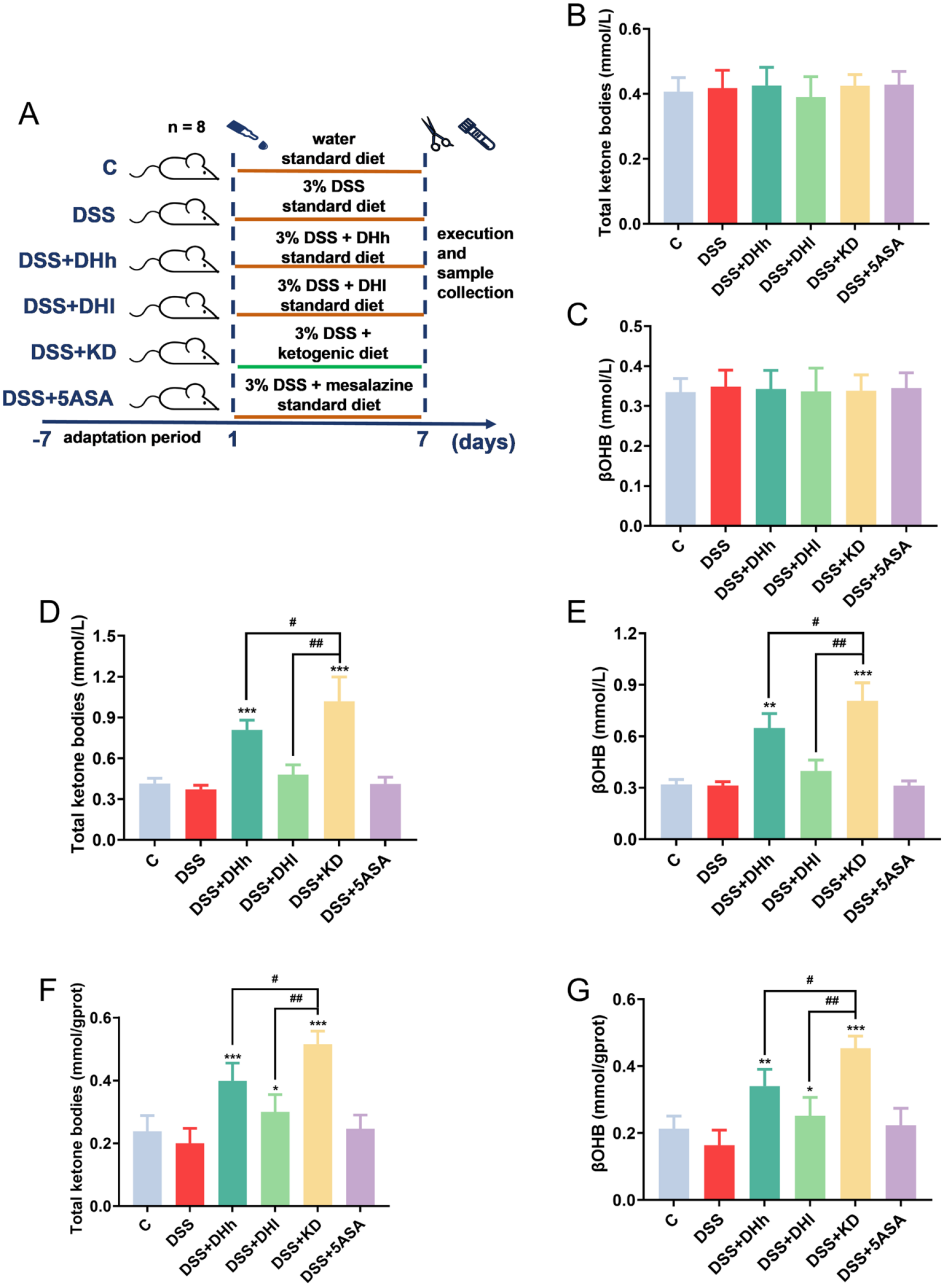
The ketogenesis in both DH and the traditional KD. (A) Animal experimental process schematic diagram; Blood total ketone bodies (B) and βOHB levels (C) on Day 0; Blood total ketone bodies (D) and βOHB levels (E) on Day 8; Colon total ketone bodies (F) and βOHB levels (G) on Day 8. ***p < 0.001, **p < 0.01, *p < 0.05 compared to the DSS group; ##*p* < 0.01, #*p* < 0.05 compared between the two groups.

To illustrate the ketogenic effects of DH and compare them with those of the traditional KD, mice were administered the traditional KD or different doses of DH extracts consecutively. Blood total ketone bodies and βOHB concentrations were tested on Day 0 (baseline) and Day 8 (after the treatment) (Figure 2B–E). Additionally, the ketone body levels in colon tissue on Day 8 were also evaluated (Figure 2F,G). The results revealed no significant differences in baseline blood ketone body levels among the groups prior to intervention. Throughout the study period, no significant differences in ketone body levels were observed among the C, DSS, and DSS + 5ASAgroups, indicating that although DSS can induce UC, it does not affect ketone bodies metabolism. Similarly, 5ASA, a commonly used medication for UC, also does not influence ketone bodies metabolism. The DSS + KD group demonstrated significantly elevated ketone body levels on Day 8 compared to the DSS group, confirming the efficacy of the KD in promoting ketone bodies production. In the DSS + DHh group, both blood and colon ketone body levels were significantly elevated compared to the DSS group, demonstrating DH's potent capacity to elevate ketone bodies in the DSS‐induced UC model.

### 3.3 Comparison of the Effects of DH and the Traditional KD on Energy Metabolism and Hepatic/Renal Function

**FIGURE 3:**
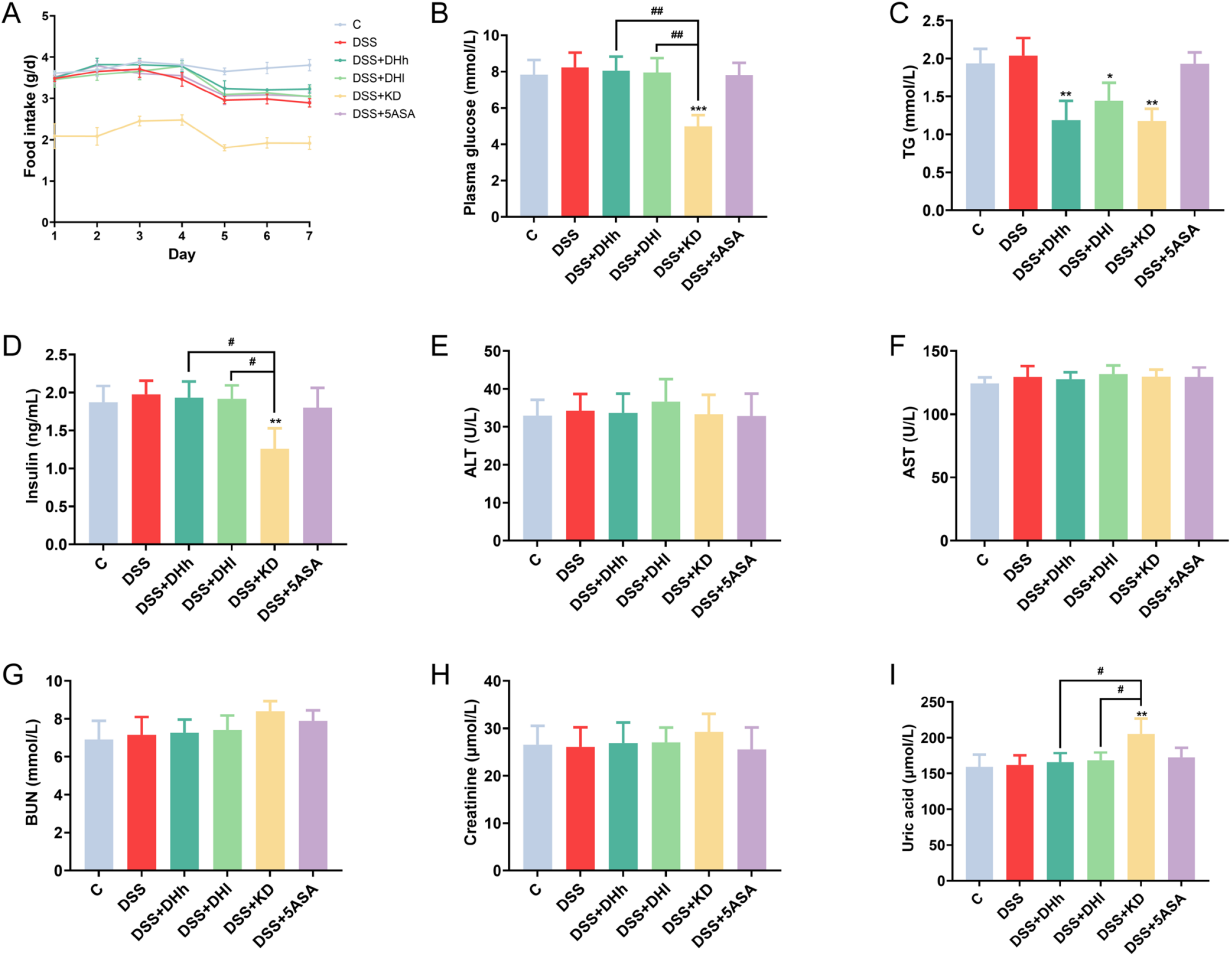
The effects of DH and the traditional KD on energy metabolism and hepatic/renal function. (A) The food intake during the experimental period; (B–D) Energy metabolism related indicators in plasma on Day 8; (E–I) Hepatic and renal function related indicators in plasma on Day 8. ***p < 0.001, **p < 0.01, *p < 0.05 compared to the DSS group; ##*p* < 0.01, #*p* < 0.05 compared between the two groups.

The food intake of mice across different groups during the experimental period is shown in Figure 3A. The DSS + KD group exhibited lower food intake compared to mice fed with standard diet, potentially due to the higher energy content per unit of the KD. On Day 8, energy metabolism related indicators revealed no significant differences between the DSS group and the C group in terms of plasma glucose, TG, and insulin levels. Conversely, the KD intervention resulted in a significant reduction in plasma glucose, TG, and insulin levels compared to the DSS group. However, the DH intervention only led to a decrease in TG levels without significantly affecting plasma glucose or insulin levels (Figure 3B–D). Regarding hepatic and renal function related indicators, including ALT, AST, BUN, creatinine, and uric acid in plasma, no significant differences were observed between the DSS group and the C group, indicating that DSS did not impact hepatic and renal function while inducing UC in this experiment. Similarly, both doses of DH did not cause significant changes in the hepatic and renal function related indicators compared to the DSS group. Nevertheless, the DSS + KD group exhibited a significant increase in uric acid levels (Figure 3E–I).

### 3.4 Comparison of the Effects of DH and the Traditional KD on UC

**FIGURE 4:**
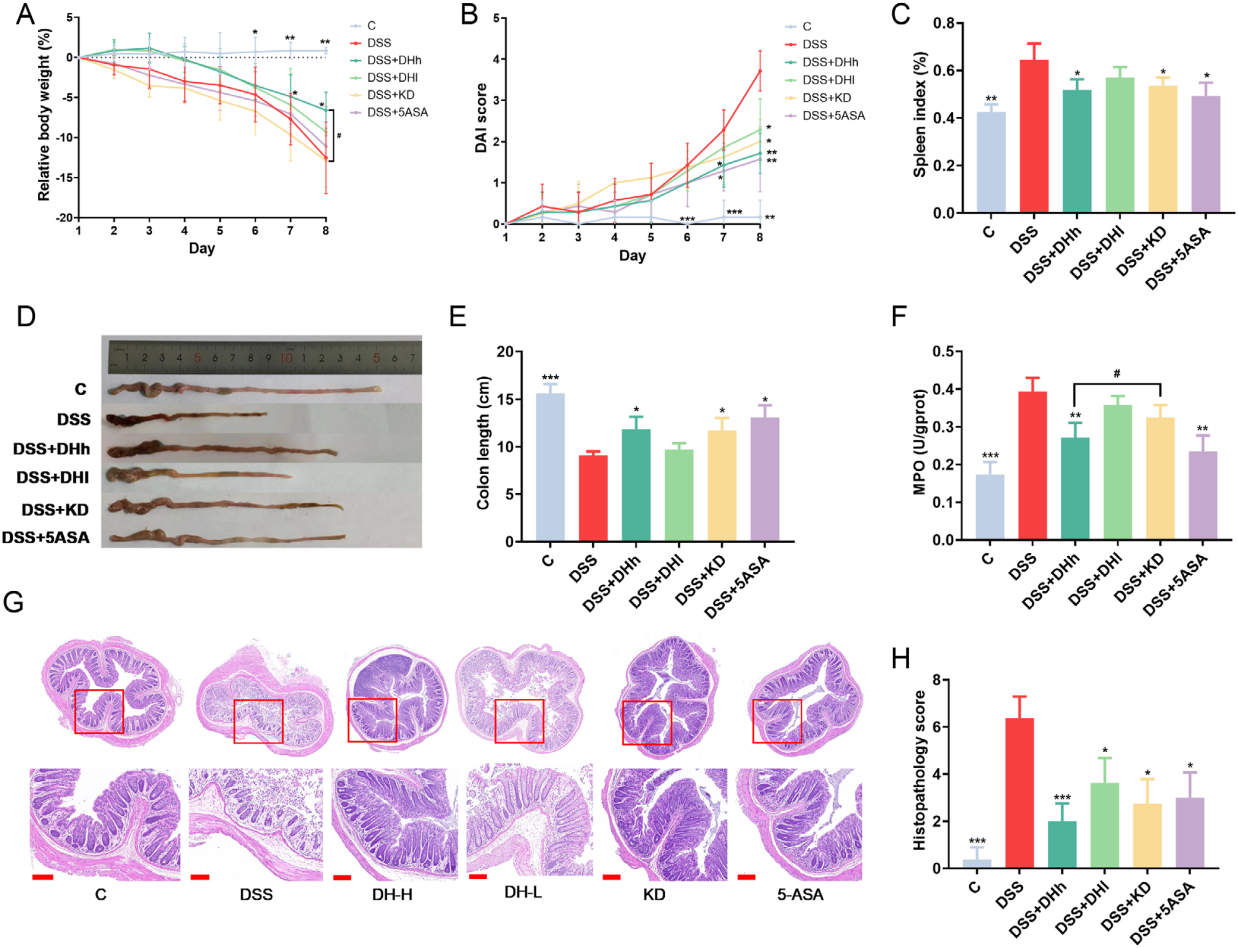
The effects of DH and the traditional KD on alleviating UC in mice. (A) The relative body weight changes compared to Day 1; (B) DAI scores; (C) Spleen index on Day 8; (D) Colon length images; (E) Colon length (from the anus to the cecum); (F) MPO levels in colon tissues on Day 8; (G) Representative images of H&E staining in colon tissues on Day 8 (scale bar: 100 μm); (H) Colon tissues histopathology scores on Day 8. ***p < 0.001, **p < 0.01, *p < 0.05 compared to the DSS group; #*p* < 0.05 compared between the two groups.

The traditional KD has been demonstrated to be effective in alleviating DSS‐induced colitis (Luiskari et al. 2024). This study similarly employed a DSS‐induced UC mouse model to evaluate the therapeutic efficacy of DH as a novel ketogenic functional food compared with the traditional KD in UC management (Figure 4). In comparison to the C group, the DSS group showed notable weight loss, increased DAI scores, and markedly elevated MPO levels. Additionally, mice in the DSS group demonstrated enlarged spleens with increased spleen index, reduced colon length, and observable mucosal damage in the colon. However, in the DSS + DHh group, interventions with DH effectively ameliorated these pathological alterations associated with UC. These findings substantiate that DH can also effectively alleviate UC in mice compared to the traditional KD.

### 3.5 Comparison of the Effects of DH and the Traditional KD on Antioxidant Stress and Anti‐Inflammation

**FIGURE 5:**
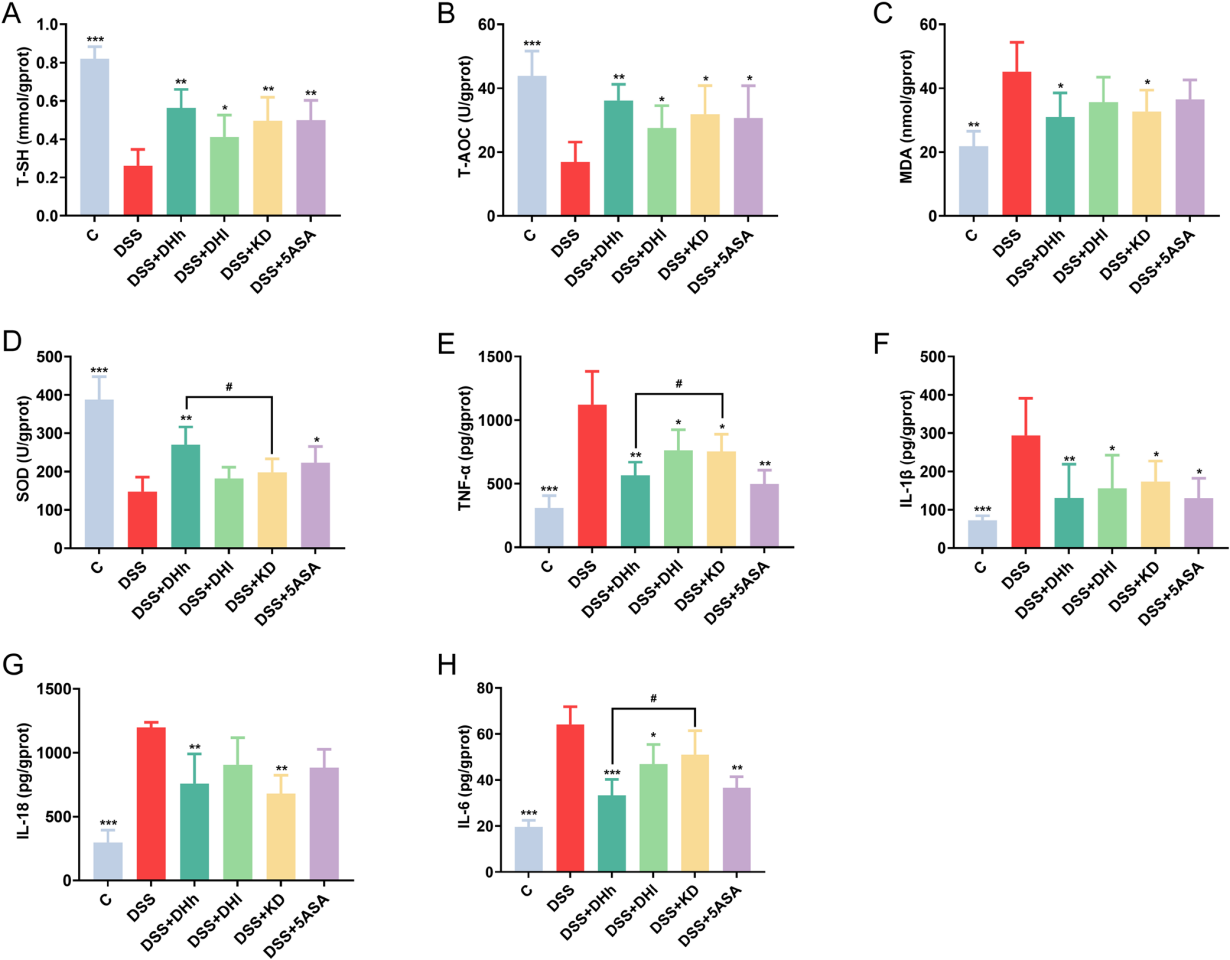
DH and the traditional KD alleviated the oxidative stress injury (A–D) and inflammatory response (E–H) in colon induced by DSS on Day 8. ***p < 0.001, **p < 0.01, *p < 0.05 compared to the DSS group; #*p* < 0.05 compared between the two groups.

One of the primary effects of the KD is the alleviation of oxidative stress and inflammation by increasing ketone bodies such as βOHB. Elevated levels of oxidative stress damage and inflammatory markers in the colon are key characteristics of UC. To compare the effects of DH and the traditional KD on oxidative stress and inflammation induced by DSS, we examined several oxidative stress related indicators, including T‐SH, T‐AOC, MDA, and SOD (Figure 5A–D), as well as inflammatory cytokines associated with colitis, including TNF‐α, IL‐1β, IL‐18, and IL‐6 (Figure 5E–H) in colon tissues. The results revealed that DSS induction led to a significant increase in MDA levels and inflammatory cytokines, while antioxidant factors T‐SH, T‐AOC, and SOD were markedly decreased. Conversely, in the DSS + DHh and DSS + KD groups, intervention with DH extracts or the traditional KD mitigated oxidative stress injury and inflammatory responses compared to the DSS group. Additionally, for certain indicators such as SOD, TNF‐α, and IL‐6, the efficacy of DH was significantly superior to that of the traditional KD.

### 3.6 DH and the Traditional KD Inhibits the Activation of the STAT3 Pathway Induced by DSS

**FIGURE 6:**
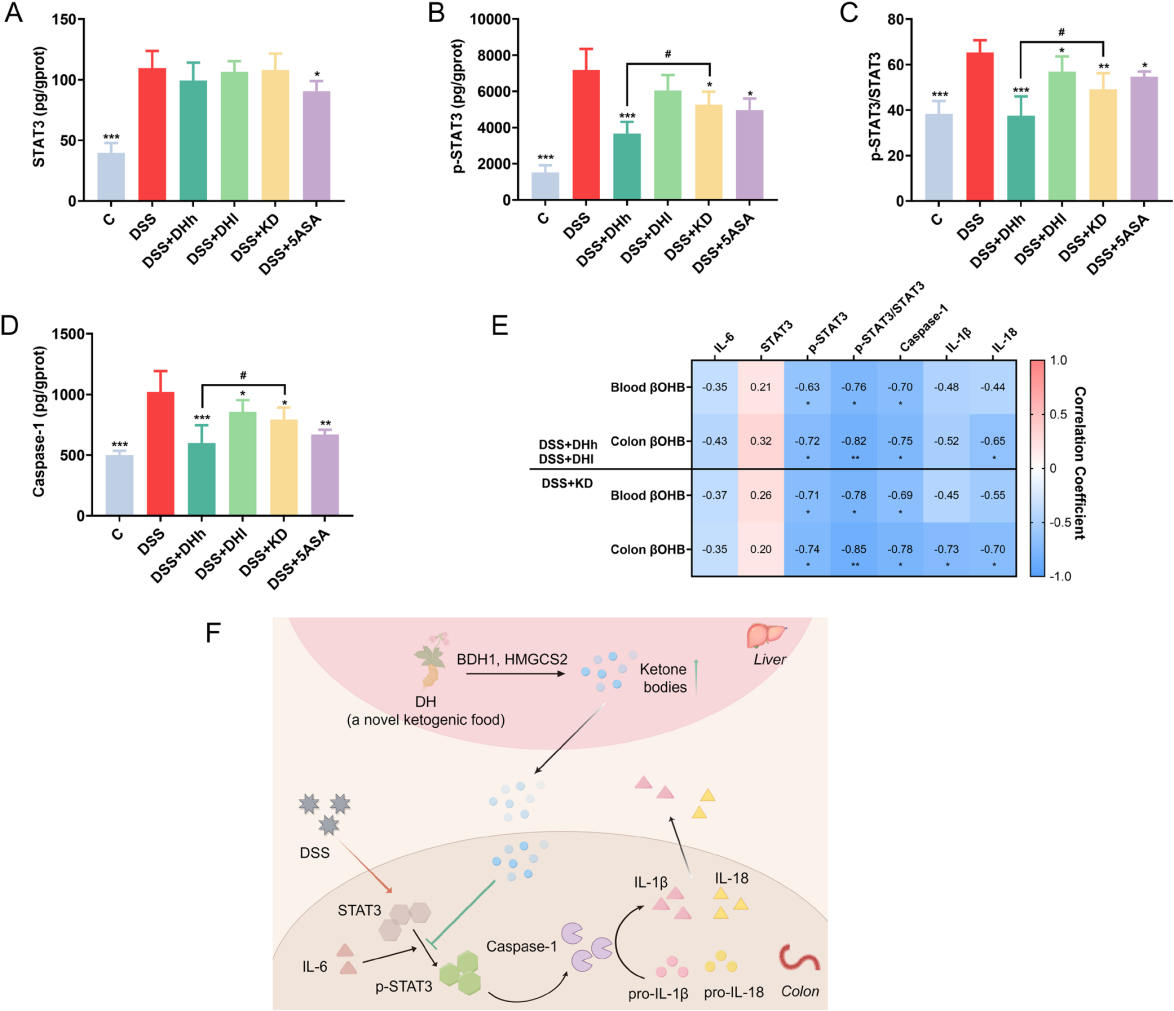
DH and the traditional KD inhibited the STAT3 pathway in UC mice. (A–D) STAT3, p‐STAT3, p‐STAT3/STAT3, and caspase‐1 levels in colon; (E) Correlation analysis between βOHB levels (in blood and colon tissues) and the STAT3 pathway in the DSS + DHh and DSS + DHl (combined), and DSS + KD groups; (F) Schematic diagram of the effects of DH on alleviating DSS‐induced UC through ketogenesis. ***p < 0.001, **p < 0.01, *p < 0.05 compared to the DSS group; #*p* < 0.05 compared between the two groups; In correlation analysis, **significance value of the correlation coefficient < 0.01, *significance value of the correlation coefficient < 0.05.

Previous studies have confirmed that ketone bodies can inhibit the STAT3 pathway to exert anti‐inflammatory effects, and the STAT3 pathway serves essential functions in UC pathogenesis (Jiang et al. 2022; Xue et al. 2026). The key nodes within the STAT3 pathway were detected, revealing significant alterations in the levels of critical proteins, including STAT3, p‐STAT3, and caspase‐1, in the DSS group following DSS induction. The positive drug 5ASA effectively inhibited the elevation of STAT3, p‐STAT3, and caspase‐1 compared to the DSS group. In both the DSS + DHh and DSS + KD groups, STAT3 levels did not exhibit significant changes compared to the DSS group, whereas p‐STAT3, the p‐STAT3/STAT3 ratio, and caspase‐1 were significantly downregulated (Figure 6A–D). Correlation analysis between βOHB levels (in blood and colon tissues) and the STAT3 pathway, as well as its downstream products IL‐1β and IL‐18 in mice, demonstrated significant correlations between βOHB levels and p‐STAT3/STAT3, p‐STAT3, and caspase‐1, particularly with the p‐STAT3/STAT3 (Figure 6E). These results substantiate that DH exhibits effects similar to the traditional KD, as both interventions modulate STAT3 phosphorylation, thereby reducing p‐STAT3 levels, the p‐STAT3/STAT3 ratio, and caspase‐1 expression, ultimately mitigating inflammatory responses.

## 4 Discussion

The KD is characterized by high fat, low carbohydrate, moderate protein, and a balanced intake of other essential nutrients. Modern studies have demonstrated that the KD has multiple applications, including anti‐epileptic effects, mitigation of metabolic diseases, and anti‐inflammatory properties (Qiu et al. 2024; Zhu et al. 2022). However, due to alterations in normal dietary structure, the application of the KD often leads to hypoglycemic reactions such as dizziness and fatigue, as well as ketoacidosis caused by excessive accumulation of ketone bodies. Prolonged use may increase the risk of hepatic, renal, and cardiovascular diseases, highlighting the importance of developing novel ketogenic approaches (Javier et al. 2024; Skartun et al. 2025). This study demonstrates that DH, a representative of MEH, can serve as a novel ketogenic functional food to enhance ketone bodies expression without altering normal dietary structure, and it can be applied to alleviate UC.

The compositional stability of active ingredients in DH is crucial for achieving reproducible experimental results. To ensure repeatability, the DH used in this study was selected as prepared slices identified in accordance with the Chinese Pharmacopeia (2025 edition). Previous studies have demonstrated that DH polysaccharides, catalpol, and ajugol exhibit both anti‐inflammatory and antioxidant properties (Bian et al. 2023; Jia et al. 2023). To ensure the quality of DH used in the study, these compounds were selected as representative active ingredients for quality control purposes. Nonetheless, more precise quality control methods, such as fingerprint profiling, are necessary to comprehensively ensure experimental reproducibility.

To investigate the mechanism underlying the ketogenic effects of DH, molecular docking approaches were used to predict the interactions between crucial ketogenesis related proteins and representative active compounds in DH. In molecular docking, a lower binding energy typically indicates a stronger binding affinity between the active ingredient and the protein. A binding energy less than 0 suggests potential spontaneous binding between the two molecules, while a binding energy less than −5.0 kcal/mol indicates effective binding (Eberhardt et al. 2021). Molecular docking analysis revealed that the binding energies of certain protein‐active compound interactions were less than −5.0 kcal/mol. Target proteins such as BDH1, HMGCS2, and ACAT exhibited high binding affinities, with binding energies less than −7.5 kcal/mol, particularly BDH1 (−9.2 kcal/mol). BDH1 is a key enzyme in ketone bodies synthesis, which regulates the synthesis and catabolism of βOHB (Ma et al. 2025). HMGCS2 catalyzes the conversion of acetoacetyl‐CoA (AcAc‐CoA) generated from β‐oxidation into 3‐hydroxymethylglutaryl‐CoA (HMG‐CoA), serving as the primary rate‐limiting enzyme in hepatic ketogenesis, and its activity significantly influences overall ketogenic metabolic flux (Puchalska and Crawford 2021). Measurement of BDH1 and HMGCS2 expression levels and enzymatic activities in mouse liver revealed that DH did not affect their expression levels but markedly enhanced the activities of these two enzymes. Based on this, we hypothesize that the ketogenic effects of DH may result from the synergistic regulatory influences exerted by a group of active components on the activities of key enzymes involved in ketogenesis. However, additional experiments are still necessary to confirm whether the compounds such as rehmannioside D, identified through molecular docking screening, are indeed active ketogenic constituents. Whether DH regulates other enzymes involved in ketone bodies synthesis (such as HMGCL) remains to be elucidated. Additionally, considering the prevalence of structurally similar compounds or unidentified compounds in natural plants, the existence of other compounds with potential ketogenic effects warrants further investigation.

To demonstrate the ketogenic effects of DH, we measured total ketone bodies and βOHB levels in mouse blood and colon tissues at the end of the experiment, while concurrently monitoring daily food intake. The results indicate that DH exerts its ketogenic effects without affecting food intake. Nevertheless, a comprehensive analysis of additional factors that may influence ketogenesis, such as caloric consumption and body composition monitoring, is necessary to further confirm the ketogenic effects. Beyond measurements at baseline and on Day 8, subsequent studies should consider incorporating intermediate assessments or dynamic monitoring of ketone levels to more accurately characterize the onset and sustainability of DH‐induced ketogenesis.

The KD has been demonstrated to be effective in the prevention and treatment of UC due to its antioxidant stress and anti‐inflammatory properties (Luiskari et al. 2024; Zhu et al. 2022). In this study, we employed a DSS‐induced UC mouse model to elucidate the differences between DH and the traditional KD in alleviating UC. The DSS‐induced UC mouse model is widely used in acute colitis research due to its high reliability, ease of operation, and broad applicability (Eichele and Kharbanda 2017). As a critical immune organ, the spleen index reflects the status of the body's immune function, providing a straightforward and sensitive approach for evaluating immune levels. MPO, a heme peroxidase primarily expressed by neutrophils, exhibits a positive correlation with the severity of UC‐related inflammation in the colon. In this study, DSS induction led to an increased spleen index and elevated MPO levels in the colon, accompanied by significant weight loss, shortened colonic length, and colonic mucosal damage, which are consistent with previous findings (Li et al. 2024; Liu, Ma, et al. 2025), confirming the successful establishment of the UC model. Interventions with DH extracts significantly ameliorated the aforementioned symptoms, indicating that DH also possesses pharmacological efficacy in alleviating UC.

Notably, throughout the experiment, mice in the DSS + KD group experienced a consistent reduction in body weight following dietary intervention, exhibiting a more pronounced decline trend compared to other groups. Concurrently, symptoms such as lethargy, hair loss, and diarrhea were consistently observed in the DSS + KD group. Measurements of energy metabolism, hepatic, and renal function related indicators revealed that the traditional KD resulted in decreased plasma glucose, TG, and insulin levels, along with elevated uric acid levels. Previous studies have demonstrated that carbohydrate restriction under the traditional KD may lead to hypoglycemia, reduced insulin levels, and a shift in lipid metabolism from synthesis and storage toward breakdown and oxidation, thereby reducing TG levels. These findings suggest that in the DSS‐induced UC mouse model, the traditional KD may present risks such as hypoglycemic reactions and hyperuricemia, consistent with the documented adverse effects associated with the traditional KD (Meng et al. 2024; Qiu et al. 2024). In contrast, the DH intervention only reduced TG levels without significant impacts on other energy metabolism or hepatic and renal function indicators that were assessed. As a classic example of the MEH, the safety profiles of oral DH administration have been extensively documented (Luo et al. 2024; Xiong et al. 2019). Therefore, in the treatment of UC, DH may offer a safer alternative for promoting ketogenesis compared to the traditional KD.

TG undergo hydrolysis to yield fatty acids, which are subsequently converted into acetyl‐CoA through β‐oxidation (Rong et al. 2024). We hypothesize that DH may enhance TG hydrolysis to generate free fatty acids, which are then subjected to β‐oxidation to produce acetyl‐CoA, a precursor for ketone bodies synthesis. Additionally, our findings indicate that DH also enhances the activity of enzymes involved in ketone bodies synthesis (such as BDH1 and HMGCS2) in the liver, thereby further promoting the conversion of acetyl‐CoA into ketone bodies. Existing studies have demonstrated that DH exerts regulatory effects on the gut microbiota (Jia et al. 2023). Given the role of the gut microbiome in ketone bodies production (Puchalska and Crawford 2021), the modulation of gut microbiota by DH may constitute a potential mechanism underlying its ketogenic effects. Moreover, previous studies have shown that the KD can also influence gut microbiota composition (Skartun et al. 2025). Given the limitation of the absence of microbiome analyses in this study, subsequent research should incorporate microbiome analyses to further evaluate the carbohydrate‐tolerant ketogenesis mechanism and safety profiles of DH as a novel ketogenic functional food.

In UC, the exacerbation of oxidative stress and inflammatory responses constitutes the principal mechanism underlying the damage to intestinal epithelial cells (Recinella et al. 2022). To further compare the antioxidant stress and anti‐inflammatory effects between DH and the traditional KD, we assessed oxidative stress markers and inflammatory factors in the intestinal tissues of mice. The results demonstrated that the oxidative stress and inflammation damage induced by DSS in the colon can be effectively mitigated by interventions with either DH or the KD.

βOHB, as the primary ketone body, has been proven to inhibit inflammatory responses mediated by STAT3 and caspase‐1, representing one of the primary mechanism by which the KD exerts its anti‐inflammatory effects (Jiang et al. 2022). STAT3 can be activated and phosphorylated into p‐STAT3 by its upstream cytokine IL‐6, subsequently exerting regulatory effects on inflammation, oxidative stress, and the immune system (Wen et al. 2024). p‐STAT3 promotes the expression of caspase‐1 by activating pathways such as the NLRP3 inflammasome and the small ubiquitin‐related modifier 1 (SUMO1) (Feng et al. 2022; Li et al. 2018). Caspase‐1 catalyzes the production of active IL‐18 and IL‐1β, which activate the downstream pathways such as NF‐κB by interacting with their specific receptors, thus inducing inflammatory responses (Winkler and Rosen‐Wolff 2015). Related studies have revealed that STAT3 and caspase‐1 play significant roles in the pathogenesis and progression of UC (Huang et al. 2024; Xue et al. 2026). In the DSS group, DSS modeling significantly increased the levels of STAT3, p‐STAT3, and caspase‐1 compared to the C group. Interventions with the traditional KD and DH extracts effectively suppressed the elevation of p‐STAT3 and caspase‐1 but showed no significant effect on STAT3. Correlation analysis revealed that on Day 8 of the experiment, βOHB levels in blood and colon tissues exhibited significant correlations with p‐STAT3/STAT3, p‐STAT3, and caspase‐1, particularly with p‐STAT3/STAT3, suggesting that both DH and the traditional KD primarily modulate the STAT3 pathway by interfering with STAT3 phosphorylation, thereby alleviating inflammatory responses. Additionally, compared to blood βOHB levels, βOHB levels in the colon exhibited stronger correlations with p‐STAT3, caspase‐1, and their downstream products IL‐1β and IL‐18. This is likely attributed to the fact that both colon βOHB and these STAT3 pathway‐related markers originate from the same tissue. However, further intervention studies and inhibitor experiments are necessary to confirm the ketogenic properties of DH can serve as a therapeutic strategy for UC by inhibiting STAT3 phosphorylation. Additionally, evaluation of upstream markers related to the caspase‐1, such as NLRP3 and SUMO1, could be conducted to elucidate the specific mechanism by which DH exerts its anti‐inflammatory effects through ketogenesis. Current studies have demonstrated that JAK2, TCR signaling, and Lck/Fyn serve as upstream regulatory factors of STAT3 phosphorylation, facilitating IL‐6 mediated STAT3 phosphorylation (Wen et al. 2024; Qin, Wang, et al. 2024). Whether the ketogenic effects of DH can inhibit STAT3 phosphorylation through upstream regulators such as JAK2 remains to be further investigated.

It is noteworthy that while the administration of DH extracts at a dosage of 1.5 g/kg/day exhibited ketogenic effects in this study, its ketogenesis was lower compared to that of the traditional KD. At this dose, however, DH exhibited more pronounced inhibitory effects on p‐STAT3 and caspase‐1 compared to the traditional KD. Measurements of oxidative stress and inflammation related markers also revealed that the 1.5 g/kg/day dose of DH showed comparable or even superior anti‐UC effects. Consequently, we hypothesize that DH mediates its therapeutic effects against UC through multiple mechanisms. Beyond its ketogenic properties, certain active components of DH, such as DH polysaccharides and catalpol, also possess antioxidant and anti‐inflammatory activities (Jia et al. 2023; Zhao et al. 2026). Moreover, DH's capacity to modulate gut microbiota may constitute an additional mechanism underlying its anti‐UC effects (Jia et al. 2023). This dual‐action synergy, combining direct phytochemical anti‐inflammatory effects with secondary ketogenic effects, further enhances DH's potential application as a novel functional food for the prevention and treatment of UC and other inflammation related diseases.

MEH offer notable advantages over currently approved therapeutic drugs in disease treatment or prevention, mainly because of their lower toxicity and fewer side effects (Xue et al. 2026). In this study, the high dose DH extracts were administered at 1.5 g/kg/day, equivalent to an estimated human equivalent dose of 0.122–0.165 g/kg/day; the calculated dose for adults (70 kg) is 8.54–11.55 g/day. In clinical practice, the oral dosage of DH typically ranges from 8 to 15 g/day, and previous studies have verified its safety when administered at standard doses for up to 8 weeks (Han et al. 2015; Wang and Xiao 2018; Zhang et al. 2022). In this study, the administration of DH at a dose of 1.5 g/kg/day effectively elevated ketone levels and alleviated UC symptoms in mice without demonstrating significant toxicity. However, further systematic evaluations, including pharmacokinetic studies, tissue distribution analyses, bioavailability assessments, and long‐term trials, are essential to comprehensively evaluate the safety and clinical translation potential of DH as a ketogenic food. It should be particularly noted that this study was conducted exclusively using a DSS‐induced UC mouse model. The ketogenic effects of DH, as well as its differences from the traditional KD in terms of energy metabolism and safety profiles, require further evaluation in healthy individuals. Therefore, further in‐depth research is essential to fully understand the pharmacological properties, safety profiles, and clinical application potential of DH as a novel ketogenic functional food.

## 5 Conclusion

This study has demonstrated that DH extracts can be used as a novel ketogenic functional food to enhance the expression of ketone bodies without altering the normal dietary structure and can be applied to alleviate UC. The potential mechanism of DH and the traditional KD in mitigating UC may both involve the inhibition of STAT3 phosphorylation. Compared to the traditional KD, DH demonstrates superior antioxidant, anti‐inflammatory, and safety profiles in the DSS‐induced UC mouse model. This study validates the potential of DH as a natural functional food ingredient for enhancing ketogenesis and treating related disorders. However, further evidence is still required to elucidate the specific mechanism by which DH exerts its carbohydrate‐tolerant ketogenesis and anti‐inflammatory effects, as well as to assess its long‐term safety profiles and clinical application prospects as a novel ketogenic food.

## Author Contributions

**Yiqiao Gao:** writing – review and editing, writing – original draft, supervision, visualization, funding acquisition, project administration, data curation, validation. **Jianhan Zhen:** visualization, software. **Yuye Yang:** investigation, formal analysis, visualization. **Simiao Wang:** investigation, methodology, software, formal analysis. **Xiangshi Song:** methodology, visualization. **Wei Tang:** methodology, formal analysis.

## Funding

This research was supported by the National Natural Science Foundation of China (No. 82304818), Natural Science Foundation of Henan (252300421139), and Key Research and Development Program of Henan (261111314600).

## Ethics Statement

All animal work was conducted in strict compliance with the Guidelines for the Care and Use of Experimental Animals issued by the Ministry of Science and Technology of the People's Republic of China (Approval No. 2006‐398), and approved by the Animal Ethics Committee of Henan Medical University (protocol code: XYLL‐20240321).

## Conflicts of Interest

The authors declare no conflicts of interest.

## Supporting information


**Figure S1:** Representative HPLC fingerprint at UV 203 nm. (A) DH extracts; (B) reference standards (a: catalpol; b: ajugol).
**Table S1:** Macronutrient percentages of both the standard diet and the ketogenic diet for mice.
**Table S2:** Evaluation criteria for disease activity index (DAI) scoring.
**Table S3:** Evaluation criteria for colon tissues histological scoring.
**Table S4:** UniProt IDs of ketogenesis‐related proteins in molecular docking.
**Table S5:** Quantitative analysis results for main active components in DH extracts.

## Data Availability

The datasets used and analyzed during the current study are available from the corresponding author on request.
