# Supplementary material for: Rehmannia glutinosa Extracts Enhances Ketone Bodies Expression and Alleviates Ulcerative Colitis as A Novel Ketogenic Functional Food
Source: Food Sci Nutr. 2026 Aug 16;14(8):e72263. doi: 10.1002/fsn3.72263 (PMC13478377; doi:10.1002/fsn3.72263)
Supplement: Supplementary file 1 [file FSN3-14-e72263-s001.docx]

**Supporting Information**

**1 Quality Control Methods for DH Extracts**

**1.1 Sulfuric Acid-phenol Method**

The polysaccharides content in DH extracts was measured using the sulfuric acid-phenol method.

DH extracts (0.10 mL) and ethanol (1.00 mL) were added together and vortex mixed. After standing for 12 h and centrifugation (4 ^o^C, 3000 g, 10 min), the supernatant was discarded. The sediment was then dissolved with 2 mL of hot water and centrifuged again. Subsequently, 1 mL of the supernatant was mixed with 1 mL of 5% phenol and 2 mL of sulfuric acid. Following incubation in a water bath for 15 min, the mixture was analyzed at a wavelength of 490 nm using a UV spectrophotometer (Shimadzu UV-2700, Japan). All organic reagents used in the experiment were purchased from Aladdin Biochemical Technology Co., Ltd. (Shanghai, China).

**1.2 Chromatographic Method**

The active compounds catalpol and ajugol in DH extracts were analyzed via high-performance liquid chromatography (HPLC).

DH extracts (50 µL) was diluted with deionized water (450 µL). After centrifugation (4 ^o^C, 3000 g, 10 min), the supernatant was separated for HPLC analysis (Thermo Fisher Dionex Ultimate 3000, USA). The analytical column (Ultimate XB-C18, 4.6 mm × 250 mm, 2.1 µm) was maintained at 25 ^o^C and the flow rate was set at 1.00 mL/min (Phase A: 0.1% formic acid, Phase B: methanol; A:B = 95:5). The UV detector was set at 203 nm. All organic reagents used in the experiment were purchased from Aladdin Biochemical Technology Co., Ltd. (Shanghai, China).

**2 The Composition of Both the Ketogenic Diet and Standard Diet**

**TABLE S1** Macronutrient percentages of both the standard diet and the ketogenic diet for mice

| **Composition** | **Quality (%)** | | **Energy (%)** | |
| --- | --- | --- | --- | --- |
|  | standard diet | ketogenic diet | standard diet | ketogenic diet |
| fats | 4.75 | 66.50 | 11.17 | 89.91 |
| proteins | 23.81 | 16.63 | 21.51 | 9.99 |
| carbohydrates | 66.37 | 0.00 | 67.32 | 0.00 |
| total energy |  |  | 3408 kcal/kg | 6657 kcal/kg |

**3 Evaluation Criteria for Disease Activity Index Scoring and Histopathological Scoring**

**TABLE S2** Evaluation criteria for disease activity index (DAI) scoring

| **DAI score** | **Weight loss (%)** | **Stool consistency** | **Occult/gross rectal bleeding** |
| --- | --- | --- | --- |
| 0 | 0 | normal | normal |
| 1 | 1-5 | soft | weakly positive hemoccult |
| 2 | 5-10 | loose stools | positive hemoccult |
| 3 | 10-20 | loose stools and adhering to the anus | visible bleeding |
| 4 | >20 | watery diarrhea | gross bleeding |

**TABLE S3** Evaluation criteria for colon tissues histological scoring

| **score** | **Inflammation** | **Mucosal damage** | **Crypt damage** | **Range of lesions (%)** |
| --- | --- | --- | --- | --- |
| 0 | none | none | 0 | 0 |
| 1 | mild | mucous layer | 0-1/3 | 1-25 |
| 2 | moderate | submucosa | 1/3-2/3 | 26-50 |
| 3 | severe | muscularis and serosa | 100% | 51-75 |
| 4 | - | - | 100% + epithelium loss | >75 |

**4 UniProt IDs of Ketogenesis-related Proteins in Molecular Docking**

**TABLE S4** UniProt IDs of ketogenesis-related proteins in molecular docking

| **Ketogenesis-related proteins** | **UniProt ID** |
| --- | --- |
| BDH1 | Q02338 |
| AAD | P23670 |
| ACAT | P24752 |
| HMGCL | P35914 |
| HMGCS2 | O14734 |

**5 Quality Control Results for DH Extracts**

**TABLE S5** Quantitative analysis results for main active components in DH extracts

| **Active components** | **Concentration** |
| --- | --- |
| polysaccharides | 225.13 ± 18.02 mg/mL |
| catalpol | 2.75 ± 0.17 mg/mL |
| ajugol | 1.56 ± 0.08 mg/mL |

**FIGURE S1** Representative HPLC fingerprint at UV 203 nm. (A) DH extracts; (B) reference standards (a: catalpol; b: ajugol)
